# Expression of a Mutant p53 Results in an Age-Related Demographic Shift in Spontaneous Lung Tumor Formation in Transgenic Mice

**DOI:** 10.1371/journal.pone.0005563

**Published:** 2009-05-15

**Authors:** Wenrui Duan, Li Gao, Xin Wu, Erinn M. Hade, Jian-Xin Gao, Haiming Ding, Sanford H. Barsky, Gregory A. Otterson, Miguel A. Villalona-Calero

**Affiliations:** 1 Comprehensive Cancer Center, The Ohio State University College of Medicine and Public Health, Columbus, Ohio, United States of America; 2 Department of Internal Medicine, The Ohio State University College of Medicine and Public Health, Columbus, Ohio, United States of America; 3 The Center for Biostatistics, The Ohio State University College of Medicine and Public Health, Columbus, Ohio, United States of America; 4 Department of Pathology, The Ohio State University College of Medicine and Public Health, Columbus, Ohio, United States of America; 5 Department of Radiology, The Ohio State University College of Medicine and Public Health, Columbus, Ohio, United States of America; 6 Department of Pharmacology, The Ohio State University College of Medicine and Public Health, Columbus, Ohio, United States of America; Ordway Research Institute, United States of America

## Abstract

**Background:**

Mutations in the P53 gene are among the most common genetic abnormalities in human lung cancer. Codon 273 in the sequence-specific DNA binding domain is one of the most frequently mutated sites.

**Methodology:**

To investigate the role of mutant p*5*3 in lung tumorigenesis, a lung specific p53(273H) transgenic mouse model was developed. Rates of lung cancer formation in the transgenic animals and their littermates were evaluated by necropsy studies performed in progressive age cohorts ranging from 4 to 24 months. In order to establish the influence of other common genetic abnormalities in lung tumor formation in the animals, K-Ras gene mutation and p16INK4a (p16) promoter methylation were evaluated in a total of 281 transgenic mice and 189 non-transgenic littermates.

**Principal Findings:**

At the age extremes of 4–12 and 22–24 months no differences were observed, with very low prevalence of tumors in animals younger than 12 months, and a relatively high prevalence at age 22 months or older. However, the transgenic mice had a significant higher lung tumor rate than their non-transgenic counterparts during the age of 13–21 months, suggesting an age-related shift in lung tumor formation induced by the lung-specific expression of the human mutant p53. Histopathology suggested a more aggressive nature for the transgenic tumors. Older mice (>13 months) had a significantly higher rate of p16 promoter methylation (17% *v* 82%). In addition, an age related effect was observed for K-Ras codons 12 or 13 mutations, but not for codon 61 mutations.

**Conclusions/Significance:**

These results would suggest that the mutant p53(273H) contributes to an acceleration in the development of spontaneous lung tumors in these mice. Combination with other genetic and epigenetic alterations occurring after the age of 13 months is intimately linked to its oncogenic potential.

## Introduction

Lung cancer is one of the most deadly cancers worldwide [1]–[3], and with more than 160,000 deaths per year. It is the number one cancer killer in the United States (**SEER** www.seer.cancer.gov; [2]). Seventy-five to eighty-five percent of lung cancers are categorized as non-small cell cancers (NSCLC); and adenocarcinoma has become the most prevalent NSCLC subtype [1], [3].

It has been reported that 50–60% of non-small cell lung cancers and 90% of small cell lung tumors contain p53 mutations; thus p53 alterations are among the most common genetic events in this malignancy [4], [5]. The majority of p53 mutations are missense and found within the sequence-specific DNA-binding domain. Codon 273 is one of the most frequently mutated sites [6]–[8]. The human mutant p53(273H), which has the most common substitution (arginine to histidine), has been shown to have both dominant-negative and gain-of-function properties [9]–[13]. Unlike most tumor-derived mutant p53 proteins, p53(273H) retains partial sequence-specific DNA-binding and transcriptional activation functions [14]–[17]. In addition, p53(273H) has also been reported to interact with and activate topoisomerase I to induce genomic instability [13], [18] and to promote the reassociation of single-stranded RNA or DNA to a double-stranded form [19]. Thus p53(273H) could conceivably lead to increased cell proliferation, aberrant DNA recombination, increased genomic instability and reduced chemotherapy efficacy [20], [21]. p53^270H/+^ mice (Murine p53 codon 270 correspond to human p53 codon 273) develop an increased incidence of carcinomas and B cell lymphomas compared to p53^+/−^ mice [22].

To study the role of p53 mutation in lung tumorigenesis, animal models in which the endogenous p53 gene is disrupted or mutated in a lung-specific manner, or in which mutant p53 transgenes are expressed in a lung-specific manner, would be of considerable value. It is of particular interest to generate lung cancer models which reflect, as closely as possible, human lung cancer development. To this end, we have developed a line of transgenic mice expressing the human p53(273H) gene under the transcriptional control of the human surfactant protein C (SP-C) promoter [23]. Human mutant p53(273H) mRNA and protein were demonstrated specifically in lung tissue [23], [24].

We previously reported the development of lung adenocarcinomas in SPCp53(273H) transgenic mice at a higher rate compared with their littermates at the age range of 13–15 months [23]. Lung cancer onset data of up to 24 months has now been collected and summarized in this report. Given their association with P53 mutations in human lung cancer, we evaluated the rates of K-ras gene mutation and p16INK4a (p16) promoter methylation of the lung tumors. γ-irradiation of tumors was performed to evaluate the functional activity of murine p53 in these spontaneous lung cancers.

## Results

### Age influences the rate of lung tumor formation

**Figure 1:**
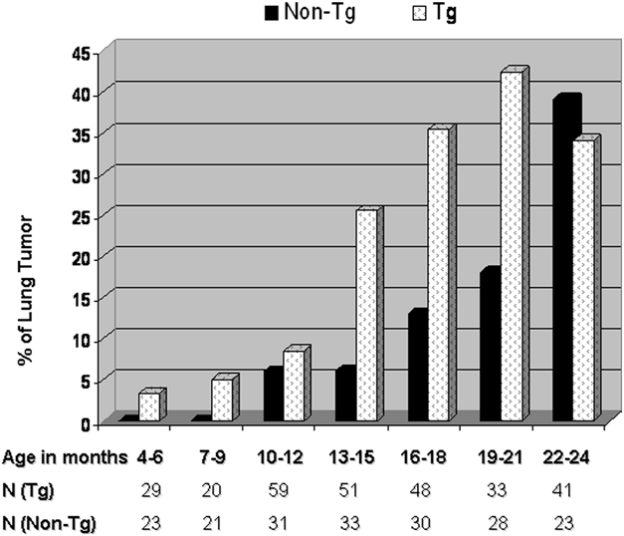
Lung tumor rate in age-matched SPC-p53(273H) transgenic mice and non-transgenic littermates. No difference was found in lung tumor rate between the transgenic mice and non-transgenic controls at the age range of 4–12 and 22–24 months. The transgenic mice (T) have a statistically significant (Fisher's exact test p<0.01) higher lung tumor rate than wildtype controls (C) during the age of 13–21 months.

Lung tumor rate and age of onset are summarized in Figure 1. Overall lung tumor rate was 23.1% (65/281) and 11.6% (22/189) in the transgenic and non-transgenic mice, respectively (Fisher's exact test p = 0.01). Peak differences in tumor rate between the transgenics and non-transgenic mice occurred during the age of 13–21 months (OR: 3.64 p<0.01). However, no significant differences in the rate of tumor development were observed at the extreme age ranges (4–12 and 22–24 months).

**Figure 2:**
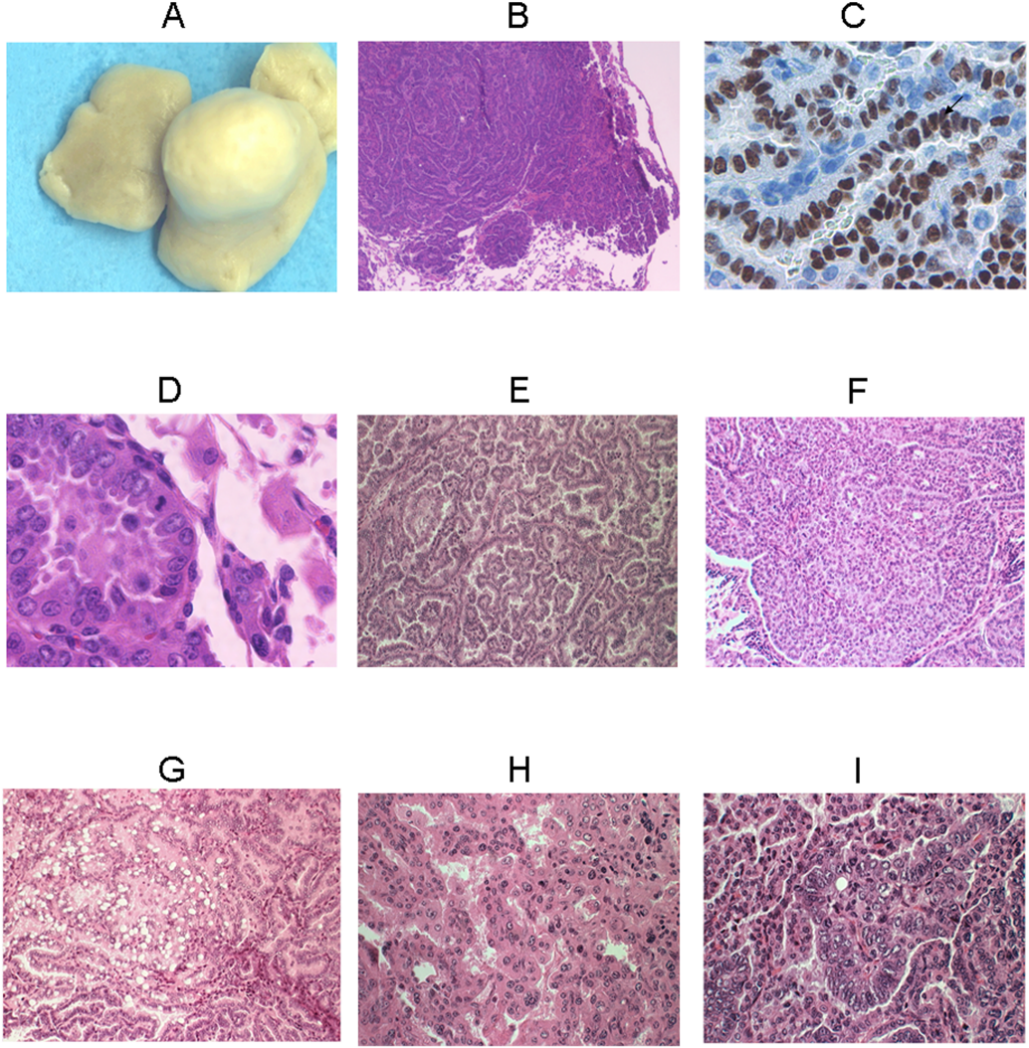
Lung tumors and characterization of the SPC-p53(273H) transgenic mice. Lung tumor from the SPC-p53(273H) mice (A). Light photomicrographs of lung tumor at 100× magnifications with HE staining (B). Immunohistochemical detection of human mutant p53(273H) with human specific p53 antibody (DO-7) in a lung adenocarcinoma from an SPC-p53(273H) transgenic mouse (C). Lung tumor cells exhibit pleomorphic nuclei at 400× magnification (D). Well-differentiated BAC/adenocarcinoma from a transgenic (E) contrasts with a BAC/adenocarcinoma showing evidence of dedifferentiation into a solid undifferentiated carcinoma (F). Other histological patterns evidencing dedifferentiation included secretory (G), oncocytic (H) and high nuclear grade (I) and were observed only in the transgenic tumors.

Histopathologically, the tumors in both transgenic and non-transgenic groups were well differentiated adenocarcinoma with a predominantly bronchioloalveolar (BAC) pattern (Figure 2). However 13 of 24 (54%) tumors examined in transgenic mice exhibited areas of variant histology, including areas of clear secretory change, areas of high nuclear grade, areas of oncocytic change and areas of solid proliferation. These variant histological patterns are evidence of dedifferentiation, a phenomenon which human lung tumors readily exhibit. In contrast only 1 of 11 (9%) tumors examined in the non-transgenic group showed evidence of dedifferentiation (Fisher's exact test p = 0.14).

### Lung tumors express type II pneumocyte differentiation

**Figure 3:**
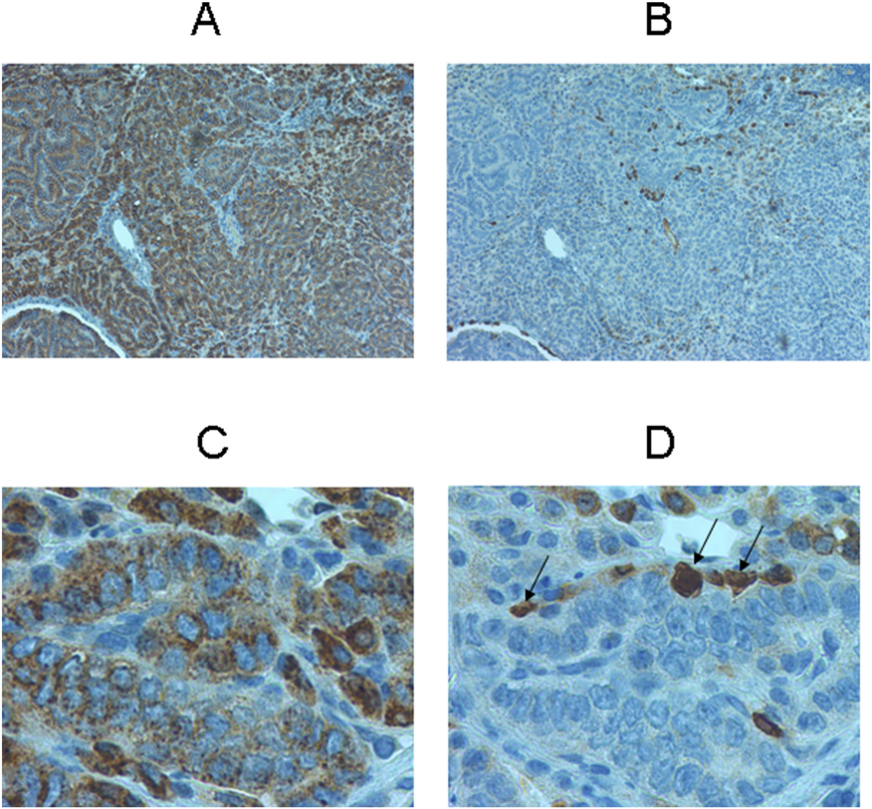
Immunohistochemical analysis of the murine lung tumor origin. A lung tumor section was stained with antibody against surfactant protein C (SP-C), commonly used as a marker for the alveolar type II cell (A). The same tumor was also stained with antibody against the Clara cell 10 protein (CC10), commonly used as a marker for Clara cell (B). The majority of the lung tumor cells were SP-C positive suggesting that these lesions arose from the alveolar type II cells (C). A small percentage of the lung tumor cells were CC-10 positive indicating of Clara cell lineage (D). The arrows pointed the CC-10 positive cells.

Immunohistochemical analysis was performed using antibodies against the surfactant protein C (SP-C) and the Clara cell 10 protein (CC10), which are commonly used markers to distinguish alveolar type II cells from Clara cells. Lung tumors collected from the transgenic mice were all SP-C positive, suggesting that these lesions expressed alveolar type II pneumocyte differentiation (Fig 3A, 3C). However, a small percentage of the lung tumor cells also expressed CC-10 protein (Fig. 3B, 3D) indicating also evidence of Clara cell differentiation. Human cancers are similarly known to express evidence of type II pneumocyte and/or Clara cell differentiation.

### Incomplete cell cycle arrest after irradiation indicates both the retention of murine wild type p53 and reduction in p53 tumor suppression function

One of the most important functions of wild type p53 is its anti-proliferation property. DNA damage up-regulates wildtype p53 and leads to cell cycle arrest which provides sufficient time for repair [25]. Ionizing irradiation induces elevation of wild type p53 protein and results in inhibition of cell proliferation, whereas knocking-out the p53 gene abolishes irradiation-induced cell cycle arrest. P53 point mutation in one allele accompanied with complete loss of the second allele of p53 is a commonly reported phenomenon in human cancers [26]–[28]. However, preservation of a wild type allele has also been reported in human neoplastic tissue [29]–[32].

**Figure 4:**
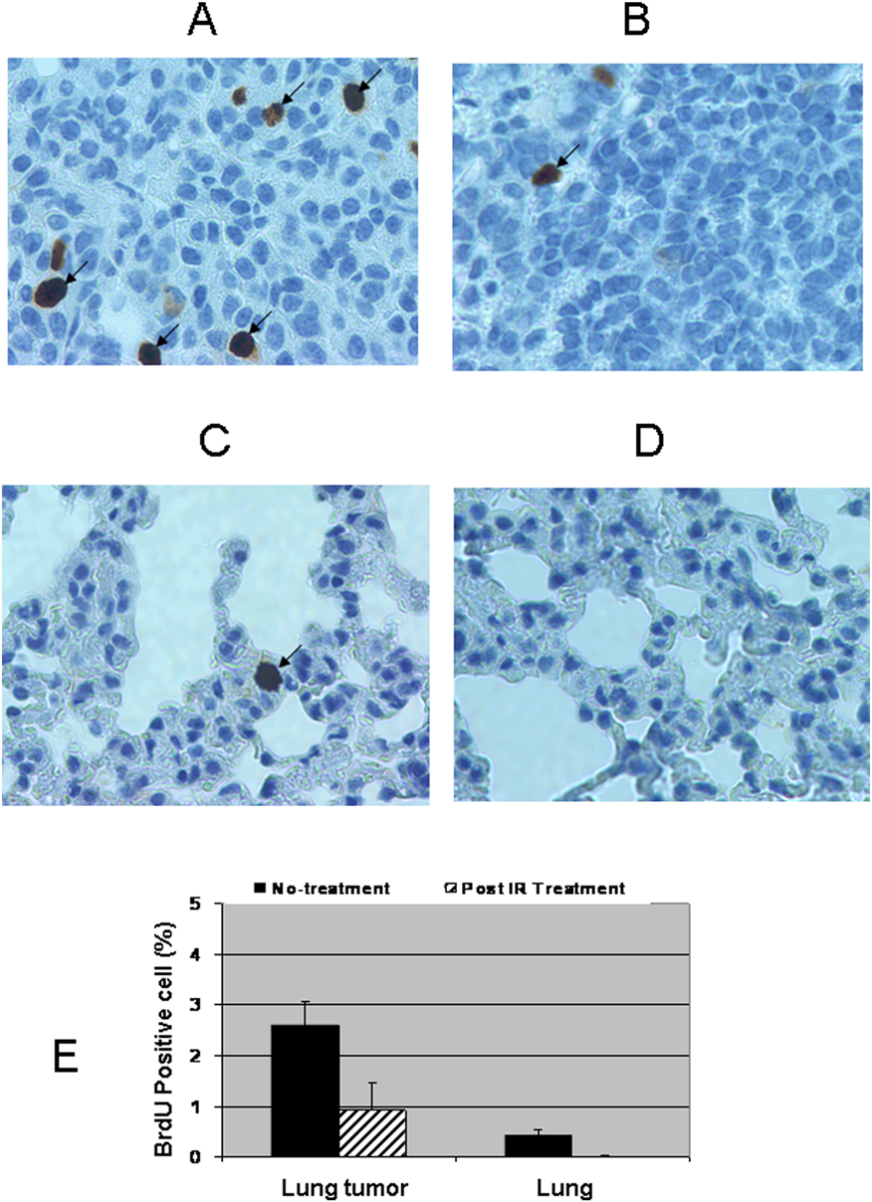
Comparison in the rate of cell proliferation between non-irradiated, post-irradiated lung tumors and normal lung tissue, determined by BrdU labeling. Mice were treated with ionizing radiation (5 Gy/mouse). 22 hours post-irradiation, mice were injected (IP) with Bromodeoxyuridine (BrdU) at a dose of 100 mg/kg in 0.9% NaCl. Lung tissue and lung tumors were harvested 2 hours after the injection, and then fixed in 10% neutral buffered formalin for 12 hours. Paraffin embedded tissue was cut and incubated with monoclonal anti-Brdu antibody (DAKO Corporation, Carpinteria, CA) for Immunohistochemical analysis. Cell proliferation in lung tumors without irradiation (A), post irradiation (B), normal lung without irradiation (C) as well as normal lung tissue post irradiation (D), were analyzed. The number of BrdU positive cell on a slide was counted directly under a compound microscope. The averaged percentage of BrdU positive cell was obtained according to thirty random images taken from each treatment and tissue type group (E). The bars show mean data ±S.D. of thirty random images from three mice per treatment. The arrows pointed the BrdU positive cells.

To evaluate whether the murine wild type p53 is intact in the lung cancers collected from the SPC-p53 (273H) transgenic mice, we treated the transgenic mice using a ^137^Cesium γ-source at a dose of 5Gy/mouse. To maximize the evaluation, *in vivo* Brdu labeling was performed. The mice were injected with Brdu at dose of 100 mg/kg 22 hr post-irradiation. Non-irradiated controls were also injected with Brdu. Both irradiated and control mice were sacrificed and lung tissues were harvested 2 hours after the Brdu injection. We analyzed 60 pathological images collected from six lung tumor bearing mice three of which had been irradiated. The rate of the BrdU positive cells was 2.61% in the non-irradiated tumors on average, whereas the proliferating rate was 0.93% in the irradiated lung tumor samples. Thus, compared to the non-irradiated tumors, the tumor cell proliferation rate was reduced 2.8 fold after γ-irradiation treatment (Fig. 4A, 4B, 4E). In the normal lung tissues, Brdu positive cell rate was 0.43% without irradiation, whereas there were almost no Brdu positive cells (0.009%) observed in the normal lung tissues post irradiation (Fig. 4C–4E).

The above results suggest that the lung tumors in the p53(273H) transgenic mice are composed of a mixed population of cells. Some lung tumor cells retain murine wild type p53, while others lost the functional murine p53 and continue to proliferate even after irradiation.

### K-ras mutation and p16INK4a (p16) methylation in the murine lung tumors

The long latency period for tumor development in the transgenic mice suggests collaboration with other oncogenic events for lung cancer development. Given the high prevalence of K-ras and p16 gene deficiencies in human lung cancers [33]–[36], we evaluated for K-ras gene mutations and p16 promoter methylation in the spontaneous lung cancers in both transgenic and non-transgenic animals.

We analyzed 28 murine lung tumors, 21 from the transgenic mice and 7 tumors from non-transgenic mice. Six of the transgenic mice were 4–12 months, and fifteen 13–24 months. All non-transgenic tumors were collected at the age of 13–24 months, given their rare occurrence at an earlier age.

It has been reported that K-ras mutations occur mainly in codons 12, 13 and 61 in murine lung tumors [37], thus we investigated the DNA sequences of exons 2 and 3 that contain codons 12, 13 and 61 using PCR amplification followed by DNA sequencing. Among 21 transgenic tumors, ten tumor samples (47.6%) contained K-ras mutations. These included four in exon 2 (codons 12 or 13) and six in exon 3 (codon 61). Among 7 non-transgenic tumors, two mutations were found in exon 2 (codon 12) and two in codon 61 (Table 1). In all cases, each tumor contained a single site mutation at the second position of the codon.

**Table 1:** K-ras mutations and p16INK4a promoter methylation in spontaneous lung neoplasm from SPC-p53(273H) transgenic mice and Non-Transgenic controls.

Cohort	Tumors analyzed	K-ras mutation		p16 methylation
		Codons 12 and 13	Codon 61	
SPC-p53(273H) 4–12 months	6	0/6 (0%)	2/6 (33%)	1/6 (17%)
SPC-p53(273H) 13–24 months	15	4/15 (27%)	4/15 (27%)	12/15 (80%)
Non-Transgenic 13–24 months	7	2/7 (29%)	2/7 (29%)	6/7 (86%)
Total	28	6/28 (21%)	8/28 (29%)	19/28 (68%)

**Figure 5:**
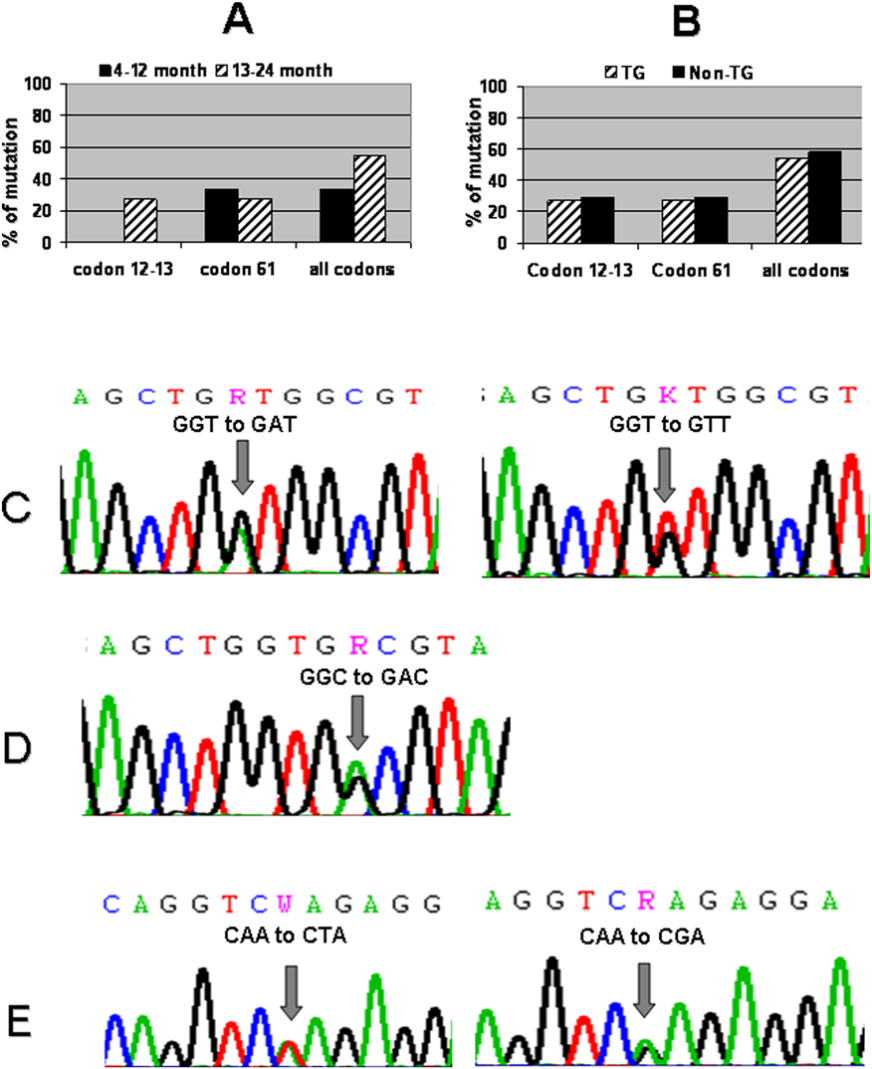
K-ras mutations in lung tumors of transgenic and non-transgenic control mice. Comparison of K-ras mutation in codons 12–13 and codon 61, (A) between 4–12 months and the 13–24 months transgenic lung tumors, and (B) between the transgenic tumors and the non-transgenic tumors in age of 13–24 months. Point mutations in (C) K-ras codon 12, (D) K-ras codon 13, and (E) K-ras codon 61.

Age differences in the type of K-ras mutations were observed, with a preference for codons 12–13 mutations to occur in the 13–24 month group (Fig. 5A) with a peak at 16–18 months (3/6 in transgenics and 5/9 in transgenic and non-transgenic tumors). In mice younger than 15 months no mutations at codons 12–13 of K-ras were found (0/10 tumors). The rate of tumors with codon 12–13 mutations was higher in the 16–18 month cohort compared to the younger age cohort in the transgenic mice (3/6 vs. 0/10, Fisher's exact test p = 0.18). Altogether (including non-transgenics) the 16–18 month cohort had a higher rate of mutations at codons 12–13 of K-ras compared to the younger age cohort (5/9 vs. 0/10). A similar rate of K-ras mutations were observed between transgenic and non-transgenic animals (Fig. 5B).

Among four mutations in codon 12, three were G to A transitions, GGT to GAT; and one was G to T transversion, GGT to GTT (Fig. 5C). Two mutations were found in codon 13; both were G to A transitions, GGC to GAC (Fig. 5D). Among eight mutations in codon 61, six mutations were A to G transitions (CAA to CGA) and two were A to T transversions, CAA to CTA (Fig. 5E).

**Figure 6:**
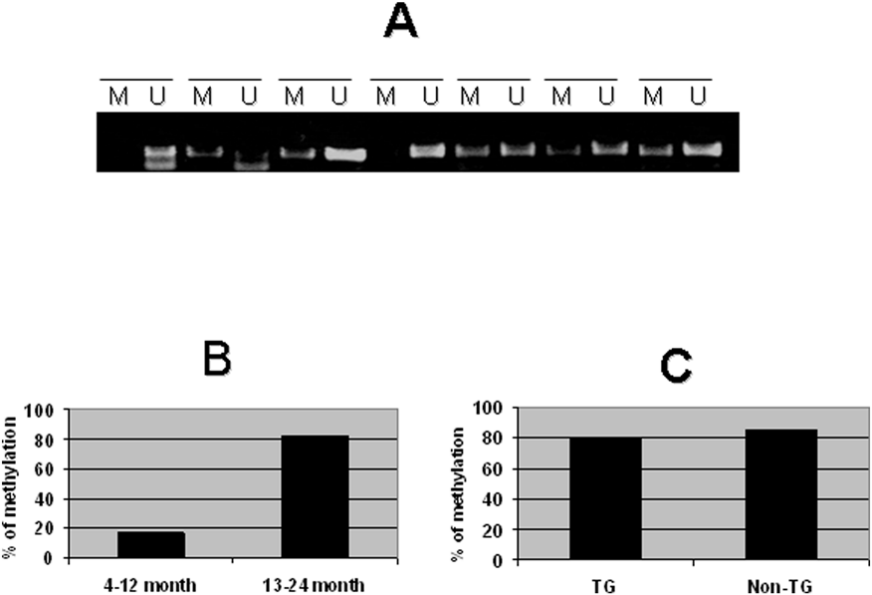
p16INK4a promoter methylations in lung tumors of transgenic and non-transgenic control mice. Bisulphite-treated tumor DNA was amplified with primers specific for either a methylated (M) or an unmethylated (U) p16INK4a promoter region (A). Comparison of p16INK4a promoter methylational rate between 4–12 months and the 13–24 months transgenic lung tumors (B), the transgenic tumors and the non-transgenic tumors in age of 13–24 months (C).

P16 promoter methylation (methylation specific PCR) was detected in 19 of 28 (68%) lung tumors (Fig. 6). Overall 13 of 21 (62%) transgenic lung tumors and 6 of 7 (86%) non-transgenic lung tumors contained p16 promoter methylation. Only one of six (17%) tumors tested at the age cohorts of 4–12 months contained p16 promoter methylation. In contrast, among the 15 transgenic tumors collected from the 13–24 month cohorts, 12 (80%) were found to have p16 promoter methylation (Fisher's exact test, p<0.1). Altogether, 18 of 22 (82%) tumors from 13–24 months (including non-transgenics) had p16 promoter methylation, which is statistically higher (Fisher's exact test, p<0.1) than for the 4–12 month group. A similar promoter methylation rate in the tumors of the transgenics and non-transgenic controls was found (Table 1).

## Discussion

The most important function of wild type p53 is the sequence specific transactivation of target genes. In normal cells, the steady state level of p53 protein is low and the half-life of p53 is very short due to the presence of negative regulators, such as Mdm2, JNK and Pirh2 [38]–[44]. However, DNA damage (e.g., radiation) induces a prominent increase in p53 protein levels [45], [46]. The increase in wild type p53 protein in response to DNA damage is believed to regulate p21 to prevent cells with genetic lesions to proliferate [47]. Either cell cycle arrest or apoptotic cell death to remove the damaged cell permanently follows [48]. Mutations of p53, the vast majority within the sequence-specific DNA-binding domain of the protein, produce mutant proteins unable to bind to and transactivate the target genes that mediate tumor suppression [48]–[50]. Restoration of p53 function leads to tumor regression in vivo [51].

The general assumption is that tumor cells with intact p53 will stop proliferating after irradiation, whereas tumor cells with deficient p53 will proliferate continually post irradiation. Our results demonstrated that some tumor cells in the transgenic animals failed to initiate cell cycle arrest post irradiation, so that 0.93% lung tumor cells were Brdu positive. In comparison, there were no Brdu positive cells observed in the normal lung tissues collected from the same mouse post irradiation. Given that even one copy of intact p53 could cause a complete cell cycle arrest in mice lungs post irradiation, it follows that the Brdu positive cells observed in the lung tumors have lost p53 function completely.

Several potential mechanisms for p53 mutant induced tumorigenesis have been proposed. One possibility is that mutant p53 inhibits the sequence-specific DNA-binding and transactivation functions of wild-type p53 in a “dominant-negative” manner by forming hetero-oligomeric complexes with it [10], [11]. Alternatively, certain p53 mutants may possess intrinsic oncogenic potential, as their introduction into cells lacking endogenous p53 has been shown to enhance the tumorigenicity of these cells [12], [50].

Since human and murine p53 proteins are fully capable of forming hetero-oligomeric complexes [52], it is therefore possible that human p53(273H) affected murine wild type p53 function in the transgenic mice studied through a “dominant-negative” effect leading to increased cell proliferation [53], [54]. Interestingly, the tumor rate differences observed between the transgenics and the non-transgenic counterparts beginning at 13 months equalizes after 22 months, suggesting that the genome instability associated with senescence [55], [56] is diluting any difference that the transgene effect could produce.

Previously we reported that the level of the mouse wild-type p53 protein was reduced in 10 of 10 tumor samples as compared to matched normal lung tissue of the transgenic mouse [57]. The reduction in the level of murine wildtype p53 protein may be associated with overexpression of p53 ubiquitin–protein ligase Pirh2 and the “dominant-negative” effect of the mutant p53.

One copy of the wild type p53 is sufficient to cause cell cycle arrest post irradiation [54], which may explain the delay in tumor development in our transgenic animals with the two murine wildtype p53 alleles in addition to the human mutant. This would also suggests that the p53(273H) has a weak dominant-negative effect. Previous in vitro studies support this notion [14], [17]. At the age of 13–21 months, when additional genetic and epigenetic alterations are able to combine with the mutant p53, a significant difference in tumor formation between the transgenic and the non-transgenic mice would then be appreciated.

We analyzed 28 spontaneous lung tumors and found that 27% of lung tumors had K-ras mutations (all somatic) at codons 12–13 at the age of 13–24 months, whereas, no mutation was detected at these codons at an earlier age. Thus the onset of K-ras mutations in codons 12 and 13 in murine lung tumor appear to be an age related phenomenon, appearing at a similar rate in tumor-developing transgenic and non-transgenic littermates.

Murine lung tumors have been shown to contain a high frequency of K-ras mutation in codon 61 [37]. This type of Kras mutation is extremely rare in human tumors, especially lung cancer. Among 28 lung tumors, eight tumors (29%) contained K-ras mutation in codon 61, and its frequency was similar between transgenics (27%) and non-transgenics (29%) and between the 4–12 months transgenic cohort (33%) and the 13–24 months transgenic cohort (27%), suggesting its lack of association with age or collaboration with P53 mutations (4–15 months vs. 16–24 months: Fisher's exact p = 0.936).

P16 gene promoter hypermethylation is a common event in lung cancers [36]; up to 80% in lung adenocarcinomas [58]. Our study demonstrated aberrant methylation of the p16 gene more frequently in the 13–24 months cohort than in the 4–12 months cohort (82% vs. 17% respectively). These results would suggest that inactivation of p16 by methylation of its promoter region is, similar to K-ras codons 12–13 mutations, a late age dependent event contributing to lung tumor formation in the p53(273H) transgenic mice.

In summary, expression of mutant p53 (273H) in mice lungs resulted in an age-related demographic shift in lung tumor formation in the transgenic mice. The latency period for tumors to develop in these transgenic mice would also suggest that mutant p53(273H) combine with other age-related genetic or epigenetic alterations, such as mutation at codon 12 or 13 of K-ras gene and inactivation of p16 by methylation of its promoter region, to promote lung tumor formation and progression in the p53(273H) transgenics. Our model may recapitulate what occurs in human cancer, where age specific differences have been reported [59], [60], and where tumor formation substantially lags behind the oncogenic stimulus [61], [62]. While p53 and K-ras codons 12/13 mutations and p16 promoter methylation status are well described in human lung cancers, the influence of age in the likelihood of these abnormalities to combine with early genetic events has not been previously explored. Our study supports the multi-step genetic hits process documented in other malignancies for which familial cancer clusters are available, and suggests the need to evaluate age related differences in the pattern of genetic and epigenetic aberrations linked to lung cancer in patient with this disease. Relevance to treatment efficacy with conventional chemotherapy or molecularly targeted treatment may be demonstrated by such studies.

## Materials and Methods

### SPC-p53(273H) transgenic and p53 knockout mice

The SPC-p53(273H) transgenic mice were generated by microinjection of a 6.7-kb fragment of the SPC-p53(273H) construct into the pro-nuclei of FVB/N mouse zygotes as previously reported [23].

All lung samples were carefully examined with the use of a dissecting microscope. All tumors observed were processed for histological analysis. In addition, 30% of lung samples with no visible surface tumors were subjected to random histological analysis. All protocols on animal subjects were approved by The Ohio State University Institutional Laboratory Animal Care and Use Committee.

### Irradiation

Total body irradiation was performed using a ^137^Cesium γ-source at a dose of 5Gy/mouse. At 24 h (hours) after irradiation, mice were sacrificed and lung tumors and normal lung tissues were harvested.

### Brdu uptake and cell proliferation indexes

Mice were injected (intraperitoneally) with Bromodeoxyuridine (BrdU) 100 mg/kg in 0.9% NaCl. Lung tumor and normal tissue were harvested 2 h after the injection, and then the tissues were fixed in 10% neutral buffered formalin for 12 h. Paraffin embedded tissue was cut and incubated with monoclonal anti-Brdu antibody (DAKO Corporation, Carpinteria, CA) for Immunohistochemical analysis. The number of Brdu positive cell on a slide was counted directly under a compound microscope (400 magnification). The average percentage of BrdU positive cells was obtained from thirty random images (three mice per group).

### Methylation Specific PCR (MS-PCR)

One microgram of genomic DNA was denatured by NaOH (final concentration, 0.275 M) for 10 min at 37°C. The denatured DNA was then treated with 10 µl of 10 mM hydroquinone, 520 µl of 3 M sodium bisulfite at 50°C overnight. The bisulfite-treated DNA was purified with Qiaquick gel extraction kit (Qiagen, Germany) according to the manufacturer's instruction. The DNA was then precipitated with sodium acetate (final concentration, 0.45 M) and isopropanol. DNA was eluted with dH_2_O and used for PCR. The primers used for the MS-PCR were (U3 = GTGATTGGGTGGGTATTGAATTTT TGTG, U4 = CACACATCATACACACAA CCCTAAACCA) for unmethylated, (M3 = CGATTGGGCGGGTATTGAATTTTCGC, M4 = CACGTCATACACACGACCCTA AACCG) for methylated, and (W1 = ACTGAATC TCCGCGAGGAAAGCG, W2 = GCACACGGCCCTGGGCCGCCG) for unmodified sequences flanking the p16 translation start site [63].

### DNA Sequencing

K-ras mutations were identified by utilizing the polymerase chain reaction (PCR) technique to amplify the second and third exons (GenBank accession NC_00072, the start codon of K-ras is located in the second exon) followed by sequence analysis of the amplified product. Primers used to amplify exon 2 were 5′- AGGCCTGCTGAAAATGACTGA (forward), 5′- TTCTTGCACCTATGGTTCCCTA (reverse). Primers used to amplify exon 3 were 5′-GGCCAGGAGTGCATTAAGAC (forward), 5′- TGCAGGCATAACA ATTAGCAA (reverse). The primers were also used for sequencing analysis.

### Histology and Immunohistochemistry

Immediately following tissue harvesting, lung tumors and normal lung tissues were placed in 10% neutral buffered formalin for 12 h, rinsed with water, and preserved in 1× Phosphate-buffered Saline (PBS). Paraffin embedded tissue was cut at 4 microns, placed on slides and stained with hematoxylin and eosin (HE). Additional sections for immunohistochemistry (IHC) were placed in a 60°C oven for 1 h, cooled, deparaffinized and rehydrated through xylene and graded ethanol solutions to water in standard fashion. All slides were quenched for 5 minutes in a 3% hydrogen peroxide solution in methanol to block endogenous peroxidase.

Antigen retrieval was performed by placing the tissue sections in a citric acid solution (Dako's Target Retrieval Solution, pH 6.1) for 30 minutes at 94°C using a vegetable steamer. Slides were then placed on a Dako Autostainer immunostaining system for IHC. Slides were blocked with 10% normal goat serum for 1 h before application of primary antibody. The detection system used was a labeled Streptavidin-Biotin Complex. Slides were then counterstained in hematoxylin, dehydrated through graded ethanol solutions and cover slipped.

The antibodies used were: anti-human p53 specific monoclonal antibody (BD PharMingen, San Diego, CA); anti-β-Actin monoclonal (Sigma, St. Louis, Missouri); anti-SP-C polyclonal (Santa Cruz, Santa Cruz, CA); anti-CC10 polyclonal (Santa Cruz, Santa Cruz, CA) and anti-Brdu monoclonal (DAKO Corporation, Carpinteria, CA).

### Statistical Methods

Given the limited number of lung tumors in certain age cohorts, age cohorts were combined into three groups, 4–12, 13–21 and 22–24 months for comparisons between transgenic and non-transgenic mice. In order to investigate how the proportion of lung tumors vary between transgenic and non-transgenic mice over age cohorts logistic regression models were used [64]. Using this model, differences between types of mice for each age cohort group were tested and odds ratios (OR) estimated through the interaction of mouse type and age cohort group. The overall comparison of tumor rates and mouse type were compared by Fisher's exact test. Multiple measurements for the % of cells BrdU positive were averaged for each mouse. Summary statistics were computed on the average of measurements. Age cohorts for K-ras mutation or p16 methylation were grouped and compared via Fisher's exact test due to the limited number of tumors. P-values were adjusted for multiple comparisons by the Holm procedure [65], [66]. Briefly the Holm procedure is a modified Bonferroni type procedure which maintains the experiment-wise error rate. Adjusted p-values were considered significant at the 0.10 level given the exploratory nature of these studies and all tests were two sided. All analyses were performed using STATA statistical software, version 10.0 [67].
